# S248. RELATION BETWEEN CHILDHOOD TRAUMA AND PSYCHOTIC SYMPTOMS IN SCHIZOPHRENIA

**DOI:** 10.1093/schbul/sby018.1035

**Published:** 2018-04-01

**Authors:** Amine Larnaout, Rahma Nefzi, Amina Aissa, Rouaa Trabelsi, Zouhaier El Hechmi

**Affiliations:** Hospital Al Razi

## Abstract

**Background:**

There is renewed interest in the relationship between early childhood trauma and risk of psychosis in adulthood. Trauma and stressful events in childhood and adolescence are known to be more prevalent among individuals with schizophrenia and other psychotic disorders than in the general population. Furthermore, other findings support the role of childhood trauma as a socio-environmental risk factor for psychotic symptoms, and research on the potential etiological relationship between trauma/stressful events in childhood/adolescence and psychotic disorders is evolving. The aim of the current study was to examine relations among all items and domains of childhood trauma and schizophrenic symptoms in patients with schizophrenia. The relationship between types of trauma and their association with psychotic symptoms was analysed.

**Methods:**

In this study, we collected data from 50 schizophrenic patients (39 males and 11 females). All patients met the DSM 5 criteria for schizophrenia. Psychotic symptoms were measured by the Positive and Negative Syndrome Scale (PANSS). Trauma and stressful events in childhood and adolescence were assessed using the Childhood Trauma Questionnaire (CTQ).

**Results:**

We found significant correlations between emotional and sexual abuse, emotional neglect and denial scale in CTQ with positive symptoms of the PANSS (p<0,05).

Meanwhile, no correlations were found between CTQ domains neither with negative symptoms nor with general psychopathology scale of the PANSS.

**Discussion:**

This study showed that childhood trauma could be a predictor factor for developing positive symptoms in schizophrenia. Most studies found similar results, showing a correlation between childhood trauma and hallucinations in schizophrenia. A correlation between childhood trauma and agressive behaviours was also described in litterature. These results went along with the stress sensitization model where the HPA axis is over-active and excessively reactive to the subsequent environemental stressors causing positive symptoms of the disease.

